# Evidence for machine learning guided early prediction of acute outcomes in the treatment of depressed children and adolescents with antidepressants

**DOI:** 10.1111/jcpp.13580

**Published:** 2022-03-15

**Authors:** Arjun P. Athreya, Jennifer L. Vande Voort, Julia Shekunov, Sandra J. Rackley, Jarrod M. Leffler, Alastair J. McKean, Magdalena Romanowicz, Betsy D. Kennard, Graham J. Emslie, Taryn Mayes, Madhukar Trivedi, Liewei Wang, Richard M. Weinshilboum, William V. Bobo, Paul E. Croarkin

**Affiliations:** ^1^ Department of Molecular Pharmacology and Experimental Therapeutics Mayo Clinic Rochester MN USA; ^2^ Department of Psychiatry and Psychology Mayo Clinic Rochester MN USA; ^3^ Peter O’Donnell Jr. Brain Institute and the Department of Psychiatry University of Texas Southwestern Medical Center Dallas TX USA; ^4^ Children’s Health Children’s Medical Center Dallas TX USA; ^5^ Department of Psychiatry and Psychology Mayo Clinic Jacksonville FL USA

**Keywords:** Depression, adolescents, machine learning, decision support tools

## Abstract

**Background:**

The treatment of depression in children and adolescents is a substantial public health challenge. This study examined artificial intelligence tools for the prediction of early outcomes in depressed children and adolescents treated with fluoxetine, duloxetine, or placebo.

**Methods:**

The study samples included training datasets (*N* = 271) from patients with major depressive disorder (MDD) treated with fluoxetine and testing datasets from patients with MDD treated with duloxetine (*N* = 255) or placebo (*N* = 265). Treatment trajectories were generated using probabilistic graphical models (PGMs). Unsupervised machine learning identified specific depressive symptom profiles and related thresholds of improvement during acute treatment.

**Results:**

Variation in six depressive symptoms (difficulty having fun, social withdrawal, excessive fatigue, irritability, low self‐esteem, and depressed feelings) assessed with the Children’s Depression Rating Scale‐Revised at 4–6 weeks predicted treatment outcomes with fluoxetine at 10–12 weeks with an average accuracy of 73% in the training dataset. The same six symptoms predicted 10–12 week outcomes at 4–6 weeks in (a) duloxetine testing datasets with an average accuracy of 76% and (b) placebo‐treated patients with accuracies of 67%. In placebo‐treated patients, the accuracies of predicting response and remission were similar to antidepressants. Accuracies for predicting nonresponse to placebo treatment were significantly lower than antidepressants.

**Conclusions:**

PGMs provided clinically meaningful predictions in samples of depressed children and adolescents treated with fluoxetine or duloxetine. Future work should augment PGMs with biological data for refined predictions to guide the selection of pharmacological and psychotherapeutic treatment in children and adolescents with depression.

## Introduction

The treatment of major depressive disorder (MDD) in children and adolescents is an important public health challenge with ongoing controversies regarding the efficacy and safety of antidepressants (Walkup, 2017). Fluoxetine and escitalopram are the only FDA approved medications for MDD in adolescents, but other selective serotonin reuptake inhibitors (SSRIs) and serotonin norepinephrine reuptake inhibitors (SNRIs) are commonly prescribed off‐label in clinical practice. Response to antidepressant therapy in children and adolescents is more heterogeneous when compared with adults (Chahal, Gotlib, & Guyer, 2020; Thapar, Collishaw, Pine, & Thapar, 2012). Major treatment planning challenges include determining which patients will benefit from acute antidepressant treatment, dosage increases, continuation treatment, and maintenance treatment.

One prior study of adolescents in treatment for MDD used logistic regression models to demonstrate that the rate of overall depressive symptom improvement was prognostic for acute treatment response (Tao et al., 2009). Recent work examined symptoms clusters in adolescents treated for MDD and identified two unique symptoms profiles. One profile demonstrated differential changes in depressive symptoms. A second profile failed to demonstrate differences among active and placebo treatments (Bondar, Caye, Chekroud, & Kieling, 2020). The findings from these two prior studies using The Treatment for Adolescents with Depression Study (TADS) data, albeit important, did not provide thresholds of improvement in specific symptoms to derive interpretable predictions of acute treatment course of MDD in young patients, nor did these studies achieve cross‐trial replications.

**Figure 1:**
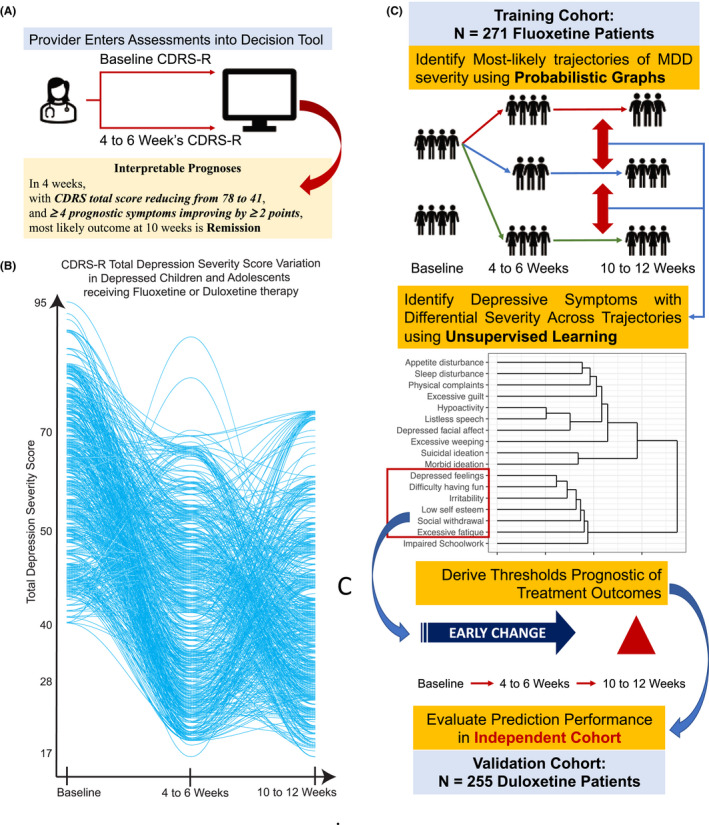
(A) Envisioned use of proposed probabilistic graph‐based tool to derive prognoses of treatment outcomes in children and adolescents treated with fluoxetine or duloxetine. (B) Trajectories of Children’s Depression Rating Scale‐Revised (CDRS‐R) total score in patients treated with fluoxetine. (C) The machine learning workflow

Interpretable outcome predictions for children and adolescents in treatment for MDD based on symptom changes would catalyze the much‐needed development of tools for shared decision making that could then be operationalized (Figure 1A) in specialty or primary care settings. These approaches are common in cardiology and other medical specialties but are underdeveloped in the child psychology and psychiatry clinical practice (Lloyd‐Jones et al., 2019). This study sought to examine probabilistic graphical models (PGMs) coupled with unsupervised machine learning techniques focused on depressive symptoms to develop accurate and valid prognostic information in children and adolescents undergoing treatment for MDD. The combination of these approaches minimizes the effects of high interindividual variability (Figure 1B) in antidepressant treatment response (Athreya et al., 2021). Unsupervised learning approaches facilitate the derivation of patient stratification and symptom clusters that are sensitive to treatment effects. Further, PGMs such as hidden Markov models (HMMs) help derive compact representations of antidepressant response to generate clinically interpretable prognoses of treatment outcomes by conditioning improvements in disease severity (Athreya et al., 2021; Ceres, Schukken, & Grohn, 2020; Goyal et al., 2018; Liu, Li, Li, Song, & Rehg, 2015; Violan et al., 2020). We hypothesized that our analytical approach (Figure 1C) would (a) identify early changes in a subset of depressive symptoms that were prognostic of eventual treatment outcomes and (b) yield improved predictions of treatment outcomes across multiple classes of antidepressants in depressed children and adolescents.

## Methods

### Datasets

TADS (March et al., 2004) and Eli Lilly Co (Atkinson et al., 2014; Emslie et al., 2014) datasets were examined in this study (Table S1 for patient characteristics). Patients received at least 10 or 12 weeks of treatment with a study drug, fluoxetine (an SSRI), duloxetine (an SNRI), or placebo. Depressive symptoms were measured using the 17‐item Children’s Depression Rating Scale‐Revised (CDRS‐R) (Poznanski & Mokros, 1996) at baseline, 4 and 10 weeks (in Eli Lilly’s datasets) or baseline 6 and 12 weeks (in TADS datasets). Early dropouts or patients in the arms of the TADS study that included cognitive behavioral therapy were not included. Each study had requisite IRB approval and clinical trial registration from respective institutions. Our current study was considered exempt by the local IRB.

PGM and prognoses rules were developed with training datasets from the TADS and Eli Lilly Co (*N* = 271) datasets (fluoxetine). The model prognostic capabilities were then examined in testing datasets from independent cohorts of patients (*N* = 255) treated for MDD with duloxetine in trials run by Eli Lilly Co (Atkinson et al., 2014; Emslie et al., 2014). Data (*N* = 265) from patients who received a pill placebo were also examined to ascertain the prognostic effects of depression symptoms that were most likely due to drug effects.

### Clinical measures and outcomes

A score of ≥ 40 on the CDRS‐R was the operational definition for MDD of moderate severity as this was the standard inclusion threshold for prior clinical trials enrolling adolescents with MDD. Remission was defined as a CDRS‐R score ≤ 28 (Atkinson et al., 2014; Emslie et al., 2014; March et al., 2004; Mayes, Bernstein, Haley, Kennard, & Emslie, 2010; Tao et al., 2009). Response was defined as a ≥ 50% reduction (or nonresponse as a lack of ≥ 50% reduction) in CDRS‐R score from baseline at 4–6 weeks or 10–12 weeks (11). If both response and remission thresholds were met, the outcome was classified as remission.

### Analytical workflow

The analytical workflow (Figure 1C) to derive predictions of antidepressant response involved five steps. Steps 1–4 used training data to identify prognostic symptoms and prognoses rules. Step 5 used the prognoses rules to derive predictions in testing data.

#### Step 1: Construction of the PGM

**Figure 2:**
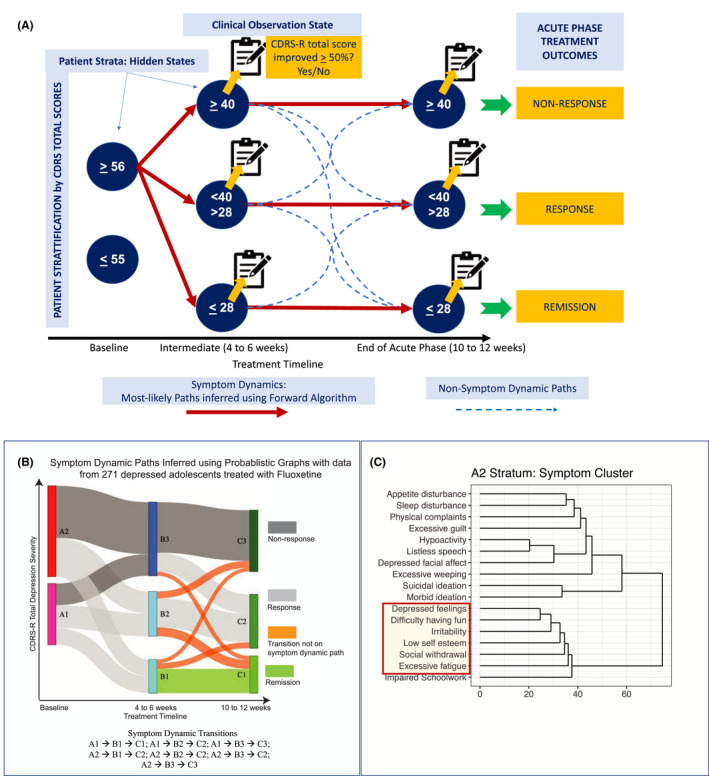
(A) Construction of the probabilistic graphical model (PGM) – a hidden Markov model comprising hidden states, observation states, and transitions. The hidden states were defined using ranges of total depression severity, wherein the ranges for depression severity at baseline were inferred using unsupervised machine learning. Observation states at the treatment’s intermediate (4–6 weeks) or endpoint (10–12 weeks) record active depression [i.e., Children’s Depression Rating Scale‐Revised (CDRS‐R) total depression severity score ≥ 40]. (B) Compact representation of CDRS‐R total score variations derived using PGM. (C) Symptom clusters of patients in A2 strata based on depression severity of CDRS‐R total scores less ≤ 55

We fitted a HMM to extract the most likely variation in depression severity during the treatment for patients starting from a given baseline strata (Figure 2A). PGMs and specifically, HMMs have provided interpretable predictions of disease trajectories in prior work (Ceres et al., 2020; Goyal et al., 2018; Liu et al., 2015; Violan et al., 2020). The HMM was characterized by (a) hidden states (patient strata defined by range of total CDRS‐R score), (b) observation states at 4–6 and 10–12 weeks (response status of patients in respective strata), and (c) forward transition probabilities (fraction of patients moving between strata of one time point to the next time point).

The baseline strata (hidden state) were characterized by multiple Gaussian curves. Gaussian mixture models algorithmically assigned patients to two strata with the ranges as follows, A1 (CDRS‐R total score ≤ 55) and A2 (CDRS‐R total score ≥ 56) (Banfield & Raftery, 1993). Clinical assessments were collected at Weeks 4–6 and Weeks 10–12 in the parent studies. Three strata were defined at treatment’s intermediate or endpoints based on symptom severity thresholds (active depression, remission, and a resulting third strata of patients with scores of CDRS‐R > 28 and < 40). The strata (with corresponding ranges of CDRS‐R total score) at Weeks 4–6 were B1 (0–28), B2 (29–39), and B3 (≥ 40). The strata (with corresponding ranges of CDRS‐R total scores) at Weeks 10–12 were C1 (0–28), C2 (29–39), and C3 (≥ 40).

All patients in B1 or C1 strata who achieved remission were also responders, 68% of patients in B2 or C2 strata were responders (without remission), and 94% of patients in B3 and C3 strata were nonresponders.

#### Step 2: Forward algorithm optimization for most likely depression severity trajectories

The forward algorithm generated likelihood for all paths originating from a baseline stratum and end in a 10–12 week strata. Forward algorithm did not condition trajectories on a specific outcome of interest (e.g., remission). Instead, the construct of a forward algorithm used data from baseline and 4–6 weeks to predict the most‐likely strata a patient will transition into at 10–12 weeks (see Appendix S1: Supplementary Methods for algorithm construction). For every pair of strata at baseline and treatment endpoint and all paths that connected them through a stratum at an intermediate time point, a path that had the highest likelihood and at least 10% of the patients from the baseline strata (Table S2) were chosen as the *symptom dynamic paths* (Figure 2B).

#### Step 3: Prognostic symptoms to predict treatment outcomes

We defined prognostic symptoms as those that met the following three criteria: (a) non‐zero symptom severities at baseline across the majority of patients (to assess the extent of early reductions in severity during treatment for predicting long‐term response; Appendix S1: Supplementary Methods), (b) similar symptom severity scores (creating symptom clusters derived using hierarchical clustering for each stratum; see Figure 2C symptom clusters for A2 stratum, see Figure S1 for A1 stratum) at all time points on symptom dynamic paths originating from a baseline stratum (to establish how many symptoms with similar severity at baseline should improve at 4–6 weeks for predicting 10–12 week outcomes), and (c) different distributions of symptom severity scores between symptom dynamic paths (to quantify the level of change in a group of symptoms at 4 weeks needed to achieve specific outcomes at 10–12 weeks). These criteria facilitated the identification of depressive symptoms with similar severities across individual time points (a grouping effect) and different levels of severity between individual symptoms dynamic pathways.

#### Step 4: Deriving predictions of acute phase treatment outcomes using early change in severity of prognostic symptoms

We first sought to derive thresholds of change (at 4–6 weeks) in prognostic symptoms that were needed to achieve one of the three categorical outcomes at 10–12 weeks. Using absolute difference in median scores of prognostic symptoms on symptom dynamic paths from baseline to 4‐ to 6‐week strata, thresholds of change needed to achieve a certain outcome of interest (Table S3) was derived. We then used chi‐square tests to identify the minimum number of prognostic symptoms needed to (or not) exceed thresholds at 4–6 weeks to be prognostic of outcomes at 10–12 weeks (Appendix S1: Supplementary Methods). For the prognoses rules associated with each baseline and 4–6 weeks transition, we calculated accuracy and odds ratio (OR) of the most‐likely outcome expected at 10–12 weeks in Table 1. Accuracy was the fraction of patients for whom the prognoses rules predicted the correct treatment outcome. The OR represents the odds that the expected treatment outcome at 10–12 weeks would occur if patients met the prognoses rule criteria, compared with the odds of the same outcome occurring in patients not meeting the prognoses rule. Statistical significance (*p*‐value) of prediction performance was derived by comparing the accuracy against the null information rate (NIR), serving as a proxy for chance. An NIR of 0.52 represents the fraction of patients in the training datasets for whom the categorical nonresponder status at 4–6 weeks correctly predicted active depression at 10–12 weeks. Finally, the Kolmogorov–Smirnov (for age) and chi‐square tests (for sex and race) were used to evaluate whether prognosis rules or accuracies were associated with age, sex, or race (the common sociodemographic factors across all datasets).

**Table 1:** (A) Prognoses performance of prognostic symptoms in patients treated with fluoxetine making specific transitions between baseline and 4–6‐week strata. (B) Prognoses performance in patients treated with duloxetine and placebo

(A) Training (TADS and Eli Lilly): Fluoxetine *N* = 271										
Baseline strata	Intermediate timepoint strata (4–6 weeks)	Number of patients making transition	Most‐likely outcome at 10–12 weeks	Prognoses rule and coverage			Probablity of most‐likely outcome (Accuracy = 100*Probabilty)	*p*‐Value of accuracy with NIR = 0.52	Odds ratio (OR)	95% Confidence interval
				Change in symptom severity (Baseline – 4–6 weeks)	Number of symptoms needing the change	Coverage (Fraction of patients covered by prognoses rule)				
A2	B3	83	Non‐response	≤2	≥4	0.80	.62	.06	12.30	2.5, 58.3
	B2	38	Response	≥2	≥3	0.92	.86	6.80E‐05	8.40	1.1, 63.7
	B1	35	Remission	≥2	≥4	0.97	.67	.09	8.00	0.78, 81
A1	B3	46	Non‐response	≤1	≥5	0.71	.66	.007	7.00	1.6, 30.4
	B2	42	Response	≥1	≥4	0.73	.84	.005	1.15	0.2, 7
	B1	27	Remission	≥2	≥4	0.77	.72	.05	8.30	1.2, 55.3

(B) Validation study								
Baseline strata	Intermediate timepoint strata (4–6 weeks)	Number of patients making transition	Most‐likely outcome at 10–12 weeks	Prognoses rule and coverage			Probablity of most‐likely outcome (Accuracy = 100*Probabilty)	*p*‐value of accuracy with NIR = 0.52
				Change in symptom severity (Baseline – 4–6 weeks)	Number of symptoms needing the change	Coverage (Fraction of patients covered by prognoses rule)		
*Testing: Prognoses performance at 4 weeks in Duloxetine treated patients (Eli Lilly N = 255)*								
A2	B3	97	Non‐response	≤2	≥4	0.85	.63	.02
	B2	38	Response	≥2	≥3	0.86	.84	6.61E‐05
	B1	23	Remission	≥2	≥4	0.78	.83	.007
A1	B3	30	Non‐response	≤1	≥5	0.80	.70	.06
	B2	39	Response	≥1	≥4	0.84	.84	6.60E‐05
	B1	30	Remission	≥2	≥4	0.77	.70	.09
								
*Testing: Prognoses performance at 4–6 weeks in Placebo treated Patients (Eli Lilly and TADS N = 265)*								
A2	B3	119	Non‐response	≤2	≥4	0.90	.58	.22
	B2	33	Response	≥2	≥3	0.93	.77	.003
	B1	18	Remission	≥2	≥4	0.88	.75	.08
A1	B3	39	Non‐response	≤1	≥5	0.62	.38	.3
	B2	33	Response	≥1	≥4	0.85	.78	.003
	B1	23	Remission	≥2	≥4	0.70	.81	.02

#### Step 5: Prediction performance in testing data

Patients in testing data were assigned to a stratum at each time point based on the mapping established in Step 1. The inputted prognostic symptom’s changes at 4 weeks established the prognoses rules established in Step 5 for the prediction of outcomes at 10 weeks.

## Results

We describe the results from each step of the analytical workflow.

### Step 1: PGM reduces the maximum number of depression severity trajectories

By stratifying patients at each time point, we expect a maximum number of MDD response 18 paths (trajectories), that is, *N* = 2 at baseline, three at 4–6 weeks and 10–12 weeks, and *t* = 3, and *N^t^* = 2*3^2^ = 18. This was a reduction in comparison with 397 unique paths without stratification (Figure 1B) – which was the motivation of Step 1 in the analytical workflow towards reducing heterogeneity of treatment outcomes.

### Step 2: symptom dynamic paths (most likely depression severity trajectories)

Of the 18 possible paths in the PGM, 7 of them had the highest likelihood scores derived from the forward algorithm in Step 2 of the analytical workflow (see Figure 2B for illustration and interpretation). Patients starting in any stratum at baseline were most likely to achieve remission (i.e., be in C1 strata) at 10–12 weeks if they transitioned into the B1 stratum at 4–6 weeks, and the clinical observation at 4–6 weeks was also remission. Patients starting in the A2 strata at baseline were most likely to achieve response at 10–12 weeks, if they transitioned into the B2 or B3 stratum at 4–6 weeks and the clinical observation at 4–6 weeks was response or nonresponse respectively; and were most likely to be nonresponders at 10–12 weeks if they transitioned into the B3 stratum at 4–6 weeks and the clinical observation at 4–6 weeks was also a nonresponse. Patients starting in the A1 stratum at baseline were most likely to be nonresponders at 10–12 weeks if they transitioned into the B2 stratum at 4–6 weeks and the clinical observation at 4–6 weeks was also nonresponse (Figure 2B). Symptom cluster derived using hierarchical clustering (Figure 2C) in A2 stratum of patients with CDRS‐R depression severity ≥ 56.

### Step 3: Prognostic symptoms

**Figure 3:**
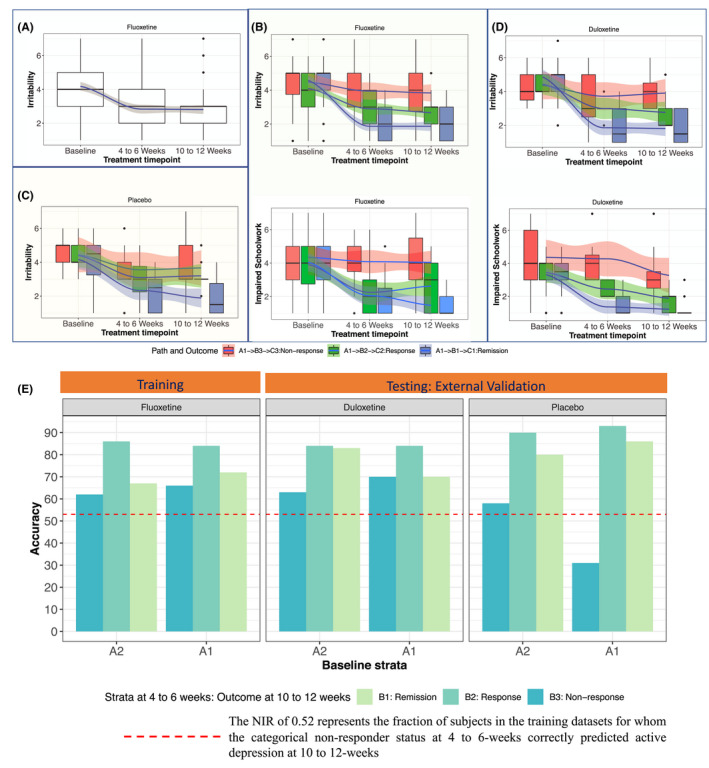
(A–D) Solid blue line was the variation in mean symptom severity, the shaded region around the solid blue line was 95% confidence interval of the variation around the mean symptom severity and the box plots visualize the variation in symptom severity at each time point. (B–D) We observe variations in symptom severity on the symptom dynamic paths originating in A1 stratum at baseline. (A) Variation in the severity of irritability in [Children’s Depression Rating Scale‐Revised (CDRS‐R) item 4] in patients originating from A1 stratum at baseline. Although the band of the shaded region was narrow around the mean indicating reduction in symptom severity in response to therapy, the height of the boxplot and the extent of whiskers indicate high degrees of variability in scores at each time point. (B and D) Visualizing variation in prognostic (irritability) and nonprognostic symptoms (impaired schoolwork) in symptom dynamic paths of patients originating in A1 stratum and treated with either fluoxetine (Figure B) or placebo (Figure C and D). (E) Accuracy of predictions derived using short‐term improvement in prognostic symptoms and CDRS‐R total depression severity

Six CDRS‐R items (difficulty having fun, social withdrawal, excessive fatigue, irritability, low self‐esteem, and depressed feelings) met the prognostic symptom criteria in the training dataset. Figures 3A,B (irritability) and S2 (all other symptoms) illustrate the variations in severity of prognostic symptoms in patients with and without the superimposition of symptom dynamic paths. In patients who were not assigned to a baseline stratum (e.g., A1 or A2), there was a high degree of interpatient variation in the irritability scores, despite a mean reduction from baseline to endpoint (as shown by the large spread of boxplots in Figure 3A). By stratifying patients and then deriving symptom dynamic paths (e.g., those originating from stratum A1, as shown in Figure 3B), the discriminatory effect of scores at 10 to 12 weeks was better reflected in the patterns of response at 4–6 weeks. No such discriminatory effects occurred for nonprognostic symptoms (e.g., impaired schoolwork) or in the prognostic symptoms (inferred from fluoxetine patients) in placebo‐treated patients (Figure 3C and Figure S3). No prognostic symptoms could be identified for patients who received placebo.

### Step 4: Prognostic performance of prognostic symptoms in training dataset

Figure 3E and Table 1A summarize the predictive performance of the changes in prognostic symptoms at 4–6 weeks from treatment initiation.

*For patients originating in the A2 stratum*: (a) the accuracy in the prediction of nonresponse at 10 to 12 weeks was 62% (OR 12.3, CI 2.5‐58.3, *p* = .06) by transitioning into the B3 stratum with ≥ 4 prognostic symptoms improved by ≤ 2 point at 4–6 weeks; (b) the accuracy in the prediction of response at 10–12 weeks was 86% (OR 8.4, CI 1.1–63.7, *p* = 6.8E−05) by transitioning into the B2 stratum with ≥ 3 prognostic symptoms improved by ≥ 2 points at 4–6 weeks; and (c) the accuracy in the prediction of remission at 10–12 weeks was 67% (OR 8, CI 0.78–81, *p* = .09) by transitioning into the B1 stratum with ≥ 4 prognostic symptoms improved by ≥ 2 points at 4–6 weeks.

*For patients originating in the A1 stratum*: (a) the accuracy in the prediction of nonresponse at 12/12 weeks was 66% (OR 7, CI 1.6‐30.4, *p* = .007) by transitioning into the B3 stratum with ≥ 5 prognostic symptoms improved by ≤ 1 point at 4–6 weeks; (b) the accuracy in the prediction of response at 10–12 weeks was 84% (OR 5.4, CI 0.2–7, *p* = .005) by transitioning into the B2 stratum with ≥ 4 prognostic symptoms improved by ≥ 1 point at 4 weeks, and (c) the accuracy in the prediction of remission at 10–12 weeks was 72% (OR 8.3, CI 0.6–7.66, *p* = .05) by transitioning into the B1 stratum with ≥ 4 prognostic symptoms improved by ≥ 2 points at 4–6 weeks.

In over 71% (Table 1) of the patients starting from any of the baseline strata, the criteria for minimum number of prognostic symptoms needed for threshold rules were satisfied. The outcome was nonresponse for nearly all (89%) of the remaining patients.

### Step 5: replication of prognostic performance of prognostic symptoms in testing datasets

Patients in the testing datasets who were treated with duloxetine were assigned to a stratum at each time point, as defined by the same range of total CDRS‐R scores derived from the training dataset. The same thresholds of prognostic symptom changes at 4 weeks derived from the training cohort were applied to the testing dataset to predict outcomes at outcomes at 10 weeks (Figure 3D and 3E, additional details in Table 1B).

*For patients originating in the A2 stratum:* (a) the accuracies in the prediction of nonresponse at 10 weeks was 63% (*p* = .02) for patients treated with duloxetine who transitioned to the B3 stratum with ≥ 4 prognostic symptoms improved by ≤ 2 point at 4 weeks; (b) the accuracies in the prediction of response at 10 weeks was 84% (*p* = 6.61E‐05) for patients who transitioned to the B2 stratum with ≥ 3 prognostic symptoms improved by ≥ 2 points at 4 weeks; and (c) the accuracies in the prediction of remission at 10 weeks was 78% (*p* = .007) for patients who transitioned to the B1 stratum with ≥ 4 prognostic symptoms improved by ≥ 2 points at 4 weeks.

*For patients originating in the A1 stratum:* (a) the accuracies in the prediction of nonresponse at 10 weeks was 70% (*p* = .06) for patients treated with duloxetine who transitioned to the B3 stratum with ≥ 5 prognostic symptoms improved by ≤ 1 point at 4 weeks; (b) the accuracies in the prediction of response at 10 weeks was 84% (*p* = 6.6E‐05) for patients who transitioned to the B2 stratum with ≥ 4 prognostic symptoms improved by ≥ 1 points at 4 weeks; and (c) the accuracies in the prediction of remission at 10 weeks was 70% (*p* = .09) for patients who transitioned to the B1 stratum with ≥ 4 prognostic symptoms improved by ≥ 2 points at 4 weeks.

Analogous to the case in the training dataset, the prognostic symptom criteria captured variations in ≥ 77% of patients from each baseline cluster across all of the testing datasets. Nearly all (93%) of the remaining patients were nonresponders at 10 weeks. Neither age, sex, nor race was associated with chances of meeting the prognostic symptom criteria or the prediction accuracy in either fluoxetine‐ or duloxetine‐treated patients.

### Exploratory analyses: prognoses performance in placebo‐treated patients

Prognostic depressive symptoms could not be identified in patients who received placebo. Table 1B summarizes the accuracy of predicting outcomes in placebo patients (assigned to baseline and 4–6 weeks strata) using the four prognostic CDRS‐R‐derived symptoms and compared these outcomes with fluoxetine treatment patients (Table 1A).

## Discussion

This study demonstrated that PGMs can generate predictions of antidepressant response in children and adolescents with MDD across two classes of antidepressants. The PGM identified seven depression severity trajectories, wherein variations of six individual depressive symptoms (difficulty having fun, social withdrawal, excessive fatigue, irritability, low self‐esteem, and depressed feelings) after 4 to 6 weeks of treatment were predictive of clinical outcomes at 10–12 weeks. The analytical approach presented in this work examined data from fluoxetine‐treated patients. A replication from duloxetine‐treated patients demonstrated accuracies of 77% (remission), 84% (response without remission), and 67% (nonresponse). The achieved accuracy and replication in prediction performance across antidepressant drug classes is an improvement over a prior reported accuracy of 72% (remission only) in fluoxetine‐treated patients with mixed linear models (Tao et al., 2009). This preliminary work suggests that computational models have future promise for assisting clinical decisions by informing physicians on the selection, use, and dosing of antidepressants for children and adolescents with MDD.

This work represents a first step in establishing a symptom‐based tool to derive interpretable predictions of treatment outcomes in children and adolescents with depression. Future efforts could integrate symptom‐based PGMs with pharmacogenomic (Ramsey, Bishop, & Strawn, 2019; Ramsey et al., 2020; Troy, Poweleit, Strawn, Martin, & Ramsey, 2020), functional connectivity (Singh, Leslie, Packer, Weisman, & Gotlib, 2018), and neural metabolomic (Gabbay et al., 2012) data to enhance the predictability of treatment outcomes. In this study, placebo response did not differentiate from antidepressant response and nonresponse to placebo did not have a predictable pattern. Further improvement in symptomatology‐based predictability of outcomes for placebo‐treated patients would be invaluable for clinical and research efforts. For example, placebo responders could be treated with, structure, watchful waiting, and psychosocial treatments could be employed first rather than an antidepressant. Conversely, patients predicted to poorly respond to placebo could receive antidepressant treatment early. Accurate predictions of placebo response in children and adolescents with MDD could also refine clinical trial methodology (Strawn & Walkup, 2020). Protocols would then specify that all placebo responders identified by PGMs exit the trial at 4 weeks. This would result in an enriched sample to examine the true effect of the active antidepressant. Early prediction and removal of placebo responders addresses a contributor to failed pharmacotherapy trials for depression in adolescents (Walkup, 2017).

The symptom trajectories identified in the study provide additional clinical translational opportunities. Future integration of neurobiological measures into our predictive model and prospective studies offers essential opportunities to study the syndromes, phenomenology, and progression of mood disorders in adolescents. Predictive symptoms from our model (difficulty having fun, social withdrawal, excessive fatigue, irritability, low self‐esteem, and depressed feelings) provide future opportunities to develop understanding of negative valence, positive valence, social processes, arousal, and regulatory systems of the Research Domain Criteria Initiative (Garvey, Avenevoli, & Anderson, 2016; Van Dam et al., 2017).

Predicting outcomes in children and adolescents treated for depression is critical in managing what could manifest into a lifelong disease burden. Recent studies raise many questions regarding the potential for over prescription, under prescription, and potential inequities of treatment for MDD in young patients (Jack et al., 2020; Sultan et al., 2018). To this end, efforts are underway to train and engage primary care physicians in the optimal treatment of MDD in children and adolescents. Algorithm‐based approaches and decision support tools informed by technological advances are understudied tools that could enhance treatment approaches for children and adolescents with depression in primary care (Blanco et al., 2017; Mann, Michel, & Auerbach, 2021). Future work should extend the use of PGMs to identify prognoses rules from the Patient Health Questionnaire modified for teens (PHQ‐9M) as this is the predominant rating scale used in primary care environments (Richardson et al., 2010).

The use of PGMs represents an analytical novelty in this work through the ability to derive interpretable prognoses of antidepressant treatment outcomes in depressed children and adolescents across two broad classes of antidepressants. The patient stratification at 4 to 6 or 10 to 12 weeks demonstrated consistency and relevance to clinical practice based on the distributions of treatment outcomes. Several prior efforts have used latent variable analyses with growth mixture models (Chekroud et al., 2017; Clapp et al., 2013; Gueorguieva, Mallinckrodt, & Krystal, 2011; Sakurai et al., 2013; Shelton et al., 2007; Smagula et al., 2015; Tokuoka et al., 2016; Uher et al., 2008, 2009) to derive trajectories of treatment response in depressed adults. Recent work also has used deep learning approaches to model disease trajectory (Zhang et al., 2019). In this context, mathematical constructs of PGMs have advantages as they infer most‐likely trajectories of disease severity by conditioning on improvements in disease severity at intermediate time points. This approach does not require domain expertise to choose and interpret paths to ensure appropriate model fitness as in the case with growth mixture models (Bauer & Curran, 2003; Gilthorpe, Dahly, Tu, Kubzansky, & Goodman, 2014; Nylund, Asparouhov, & Muthén, 2007; Tu, Tilling, Sterne, & Gilthorpe, 2013; Wills, Silverwood, & De Stavola, 2014). The mathematical formulation presented in this work can be expanded to include extended study durations and asynchronous time points by modeling the PGM as a Markov jump process (Wang, Sontag, & Wang, 2014).

It is noteworthy that age, sex, and race did not impact prognostic symptom criteria thresholds nor prediction accuracy our model in patients treated with fluoxetine or duloxetine. This finding contrasts with prior work (Mayes et al., 2007; Strawn et al., 2021). Future research should focus on this finding to develop the current predictive model as neurobiological biomarkers are incorporated. With respect to clinical translation and implementation, a decision support tool that does not consider age, sex, nor race would have both disadvantages and advantages.

There are important limitations to consider for the interpretation of the current findings. Future work will need to explore additional categorizations of the CDRS‐R at baseline and intermediate time points would enhance the predictive performance of our model. For example, more categories or different categorizations might yield superior predictive performance. Early prediction windows such as 1 to 2 weeks or long‐term outcomes such as 6 months would have clinical utility, but this was not possible due to the structure of the training and replication datasets. As the PGM utilized data from baseline, intermediate and endpoint visits of the treatment, we did not use data from patients who dropped out in the respective clinical trials. Hence, the current findings do not account for variations in antidepressant class, dosing, patient dropouts, or variable dropout rates across studies. As such this limitation is not specific to this work but is common in all machine learning based approaches to predict outcomes (data derived after study completion) in clinical psychiatry (Chekroud et al., 2016, 2017; Iniesta et al., 2016, 2018; Koutsouleris et al., 2016). However, recent work with Bayesian hierarchical modeling suggests that antidepressant class and dose are important considerations (Suresh, Mills, Croarkin, & Strawn, 2020). The focus of this study was to establish predictability of response to pharmacotherapy for depression in child and adolescents. The methodology used in this work could however be applied to extracting response trajectories to CBT or sequential CBT combined with pharmacotherapy. Several other factors that are not considered in this work include duration of illness, adherence, family function, socioeconomic status, comorbidities, and family history of psychiatric illness. However, given the nature of the clinical trials represented in the datasets, certain assumptions are tenable. The patients were not actively using alcohol or street drugs. The children and adolescents and families were able to participate in structured follow‐up appointments and assessments as dictated by the respective study protocols. Further, the patients in the current study had a valid and reliable diagnoses of MDD, did not have treatment resistant depression, and were not psychotic. We did not have data on pubertal status and could not assess its impact on prognoses performance. While the consistent prediction performances across completers of respective studies is a strength of this work, it is not possible to rule out subtle effects of adverse reactions, dropout rates, comorbidities, or additional demographic characteristics which we did not account for. Finally, our approach relies on the acceptance of exchangeability assumptions as multiple observations were pooled across clinical trial datasets (Cole & Frangakis, 2009).

## Conclusion

This study provides evidence that PGMs coupled with unsupervised machine learning techniques provide clinically relevant predictive tools for children and adolescents with MDD treated with fluoxetine or duloxetine. Future efforts should examine prospectively treated patients, account for antidepressant class, examine dosing, consider psychotherapy, and integrate potential biomarkers. Finally, to further improve predictability and replicability of treatment outcomes across treatment classes beyond antidepressants, symptom‐based PGMs could be integrated with pharmacogenomic, functional connectivity, and neural metabolomic data.

## Supporting information


**Appendix S1.** Supplementary methods.
**Figure S1.** Symptom cluster in A1 stratum.
**Figure S2.** Illustrating the variations of prognostic and nonprognostic symptom’s severity of CDRS‐R scale on symptom dynamic paths originating from A1 stratum at baseline in patients treated with fluoxetine.
**Figure S3.** Illustrating the variations of prognostic and nonprognostic symptom’s severity of CDRS‐R scale on symptom dynamic paths originating from A1 stratum at baseline in patients treated with placebo.
**Table S1.** Sample sources and characteristics.
**Table S2.** Path likelihoods.
**Table S3.** Median symptom severity scores on symptom dynamic paths.
