# Supplementary material for: Evidence for machine learning guided early prediction of acute outcomes in the treatment of depressed children and adolescents with antidepressants
Source: J Child Psychol Psychiatry. Author manuscript; Available in PMC 2022 Nov 1. (PMC9475486; doi:10.1111/jcpp.13580)
Supplement: Supplementary file 1 [file NIHMS1795612-supplement-Supplementary_Materials.docx]

**SUPPORTING INFORMATION**

**Evidence for Machine Learning Guided Early Prediction of Acute Outcomes in the Treatment of Depressed Children and Adolescents with Antidepressants**

Arjun P. Athreya,^1^ Jennifer L. Vande Voort, MD,^2^ Julia Shekunov,^2^ Sandra J. Rackley,^2^ Jarrod M. Leffler,^2^ Alastair J. McKean,^2^ Magdalena Romanowicz,^2^ Betsy D. Kennard,^3^ Graham J. Emslie,^3,4^ Taryn Mayes,^3^ Madhukar Trivedi,^3^ Liewei Wang, ^1^ Richard M. Weinshilboum,^1^ William V. Bobo,^5^ Paul E. Croarkin,^2^ 

^1 ^Dept. of Molecular Pharmacology and Experimental Therapeutics, Mayo Clinic, Rochester, MN, USA.

^2^ Dept. of Psychiatry and Psychology, Mayo Clinic, Rochester, MN, USA.

^3^ Dept. of Psychiatry, Univ. of Texas Southwestern Medical Center, Dallas, TX, USA.

^4^ Children’s Health, Children’s Medical Center, Dallas, TX, USA

^5^ Dept. of Psychiatry and Psychology, Mayo Clinic, Jacksonville, FL, USA.

**APPENDIX S1. SUPPLEMENTARY METHODS**

**Analysis Workflow**

A machine learning workflow comprising 5 steps was developed to predict eventual treatment outcomes using a set of individual depressive items with homogeneity in their longitudinal response. 

*Step -1 Construction of the PGM***:** Unsupervised learning (specifically, Gaussian mixture models) was used to infer patient subgroups at baseline, as described in prior work with depressed adults(1). Gaussian mixture models were chosen because of inherent latent structures in the distribution of depression severity scores (i.e., the distribution of scores was likely characterized by multiple Gaussian curves). Inputs to the Gaussian mixture models were CDRS-R total scores baseline in patients treated only with fluoxetine. Using this algorithmic formulation, two strata of patients (patient clusters) were inferred in the training datasets based on total CDRS-R scores at baseline. The letters (e.g., A, B, and C) represent the treatment time-points  and the numeric suffix at each time-point represents the level of depression severity, with “3” being the most severely depressed patients and “1” being the least-severely depressed. The ranges of total CDRS-R scores for each cluster are shown below: 

Baseline stratifications: A1 [≤55], A2 [≥ 56];

Week 4-6 stratifications: B1 [0–28], B2 [29–39], B3 [≥ 40]; and 

Week 10-12 stratifications: C1 [0–28], C2 [29–39], and C3 [[≥ 40]. 

*Step -2 Forward Algorithm Optimization for Most Likely Depression Severity Trajectories:* Probabilistic graphs (expressed as a hidden Markov model) with forward algorithm were used to explore all possible paths connecting a given baseline cluster to patient clusters at 4 to 6 weeks, and then to 10 to 12 weeks. The likelihood of each path was computed using the recursive forward algorithm by defining the graph as a hidden Markov model (2). The “most likely” paths for each of the pairs of baseline and 10-12 week clusters (e.g., A1, C1) were chosen based on their having the highest likelihood scores, provided they had at least 10% of patients of the cluster from which patients originated. These unique “most likely” paths were subsequently referred to as *symptom dynamics paths*.

The forward algorithm is a recursive algorithm to compute the likelihood of a path given a starting state, transition probabilities (from previous hidden state to current hidden state), observation probabilities for a given hidden state, and path probability until reaching the current state. In the forward algorithm formulation ($P_{O}(C_{t})=  \sum_{t∈T}{p(O\vert C_{t})P_{O}(C_{t-1})p(C_{t-1}→C_{t})}$) for computing the path probability at a cluster *C*_*t*_ in time *t* and a given observation *O*, , $p(O\vert C_{t})$ is the probability of observation O in *C*_*t*_*,* $P_{O}(C_{t-1})$ is the probability of path until reaching *C*_*t*_ from cluster *C*_*t-1*_ at time t-1, and $p(C_{t-1}→C_{t})$ is the probability of transition from cluster *C*_*t-1*_ *to C*_*t*_. 

A plain language example of this formulation used this work is as follows: Let us suppose that we have a path from A2 (baseline) 🡪 B3 (4 to 6 weeks) 🡪 C3 (10 to 12 weeks), having observed non-response at B3 and C3. We first compute the path probability of patients traversing from A2 🡪 B3 and observing non-response at B – in this case  $p(C_{t-1}→C_{t})$ is the fraction of patients traversing from A2 🡪 B3 (from baseline (t-1) to 4 to 6 weeks(t)), and $p(O\vert C_{t})$ is the probability of non-response at B3 among patients who have traversed from A2 to B3. Then, we want to compute the path probability in reach C3 via B3, having originated from A2. Now, $p(C_{t-1}→C_{t})$ is the fraction of those who transition from B3 to C3 having originated at A2, $p(O\vert C_{t})$ is the probability of non-response among patients in C3 who have transitioned from A2 🡪 B3, and $P_{O}(C_{t-1})$ is the probability computed from first transition (A2 🡪 B3) and having observed non-response as the outcome at B3. This forward algorithm computation can continue beyond 3 time-points. 

*Step - 3 Prognostic Symptoms to Predict Treatment Outcomes:* To extract homogeneous patterns of antidepressant response, “prognostic depressive symptoms” were defined using three criteria: 1) similar response patterns at all time-points, 2) low inter-individual variability, and 3) patterns of change that were statistically distinct within each of the symptom dynamic paths (inferred in Step - 1 using total depression severity scores). First, unsupervised machine learning (hierarchical clustering with complete linkage) was used to identify individual CDRS-R scale items with similar rating patterns (clustered together with a common parent in the tree, except the common parent of the entire hierarchy) within the patient clusters at baseline, 4 to 6 weeks, and 10 to 12 weeks. Second, we identified symptom clusters wherein clinician ratings for each of the scale items at baseline had a nonzero median and low inter-individual variability. A given item was defined as having low inter-individual variability if the chi-square test for the distribution of clinician ratings was significant after correcting for multiple comparisons (p-value divided by total number of symptoms), with the null hypothesis being that the distributions of ratings for that item were equal. Third, the Kolmogorov-Smirnov test was used to determine if there were statistically significant differences in the distributions of prognostic symptom scores at 4 to 6 weeks between each of the symptom dynamic paths leading to non-response, response, and remission at 8 to 12 weeks, from a given baseline cluster. The variation in these prognostic symptom’s scores within specific symptom dynamic paths were visualized using average smoothing kernels.

Assessing Antidepressant Effects on Prognostic Depressive Symptoms: The Mann-Whitney U-test was used to assess whether the severity of the prognostic depressive symptoms (expressed as a rank order) changed significantly as a likely response to antidepressant treatment between two consecutive time-points on a given symptom dynamics path. The rank order test was constructed as follows. Rank order test construction: For example, consider a pair of clusters from consecutive time-points (A1 and B1) on a given path (A1 🡪 B1 🡪 C1), and a specific item from the CDRS-R (e.g., irritability). We identified placebo-pill treated subjects on the same path (i.e., assigning patients to the clusters with same depression severity range as citalopram/escitalopram treated patients). Then we tested if the clinician ratings of severity of the same item were significantly different between patients in the B1 cluster of patients treated with citalopram/escitalopram and those of placebo-pill treated patients. If the p-value (with Bonferroni correction for multiple comparisons due to multiple prognostic symptoms) was significant, then we conclude that the changes in clinician ratings of depressive items’ observed severity were more likely due to antidepressants than to chance. 

*Step – 4 Deriving Predictions of Acute Phase Treatment Outcomes Using Early Change in Severity of Prognostic Symptoms:* This step defined the minimum number of prognostic symptoms and levels of improvement in the prognostic symptoms needed at 4 to 6 weeks (given a specific baseline cluster) to achieve specific outcomes at 10 to 12 weeks. First, the threshold of improvement/failure to improve was chosen based on changes in median scores on symptom dynamic paths between a baseline and 4 to 6 -week cluster. Second, a chi-square test was conducted on a table comprising the number of prognostic symptoms that exceeded (or failed to exceed) the threshold at 4 to 6 weeks, versus the outcome labels (e.g., remitters vs. non-remitters, or responders vs. non-responders) at 10 to 12 weeks. If the chi-square test’s p-value was significant for remission or response/non-response, we computed the probability of the outcome based on how many symptoms had to exceed (or failed to exceed) the threshold. If the p-value was not significant, no conclusions about treatment outcome based on changes in any number of prognostic symptoms were possible. We then computed the accuracy and odds ratio (OR) of the most-likely outcome expected at 10 to 12 weeks in patients who transitioned from a baseline stratum to a stratum at 4 to 6 weeks. The OR (and associated p-value) represents the odds that the expected treatment outcome at 10 to 12 weeks will occur if patients are covered by the prognoses rule, compared to the odds of the same outcome occurring in patients not covered by the prognoses rule.

*Step – 5 Prediction Performance in Testing Data:* We used the prognoses rules derived from fluoxetine treated subjects in TADS and Eli Lilly to derive the prognoses in duloxetine and placebo treated patients (independent samples from fluoxetine treated patients). 

**SUPPLEMENTARY FIGURES** 

**Figure S1.** Symptom cluster in A1 stratum. 

**Figure S2.** Illustrating the variations of prognostic and non-prognostic symptom’s severity of CDRS-R scale on symptom dynamic paths originating from A1 stratum at baseline (i.e., A1 🡪 B1 🡪 C1, A1 🡪 B2 🡪 C2 and A1 🡪 B3 🡪 C3) in patients treated with fluoxetine. 

**Figure S3.** Illustrating the variations of prognostic and non-prognostic symptom’s severity of CDRS-R scale on symptom dynamic paths originating from A1 stratum at baseline (i.e., A1 🡪 B1 🡪 C1, A1 🡪 B2 🡪 C2 and A1 🡪 B3 🡪 C3) in patients treated with placebo.  The prognostic symptoms are those derived using data from patients treated with fluoxetine.

**Figure S1**

**Figure S2**

**Figure S3**

**SUPPLEMENTARY TABLES**

**TABLE CAPTIONS**

**Table S1.** Sample Sources and characteristics. Treatment for Adolescents with Depression Study (TADS) is a study conducted with trial identifier NCT00006286, HMCK and HMCL are studies conducted by Ely Lilly and Company under trial identifiers NCT00849901 and NCT00849693 respectively. 

**Table S2.** For each path we illustrate the path likelihood used to identify symptom dynamic paths. Symptom dynamic paths between a baseline and 10 to 12week strata are highlighted in green based on highest likelihood score. For example, the symptom dynamic path between A1 and C1, is A1 🡪 B1 🡪 C1 as the path has likelihood greater than other paths between A1 and C1. The ranges of depression severity scores in each stratum are as follows: A1 [≤ 55], A2 [≥ 56]; B1 [≤ 28], B2 [≥29 and ≤39], B3 [≥ 40] and C1 [≤ 28], C2 [≥29 and ≤39], C3 [≥ 40]. 

**Table S3.** Median symptom severity scores on symptom dynamic paths.

**Table S1.** 

**Table S2.** 

**Table S3.**

 

**REFERENCES**

1.  Athreya A, Iyer R, Neavin D, Wang L, Weinshilboum R, Kaddurah-Daouk R, Rush J, Frye M, Bobo W. Augmentation of Physician Assessments with Multi-Omics Enhances Predictability of Drug Response: A Case Study of Major Depressive Disorder. IEEE Comput Intell Mag. 2018;13:20-31.

2.  Athreya AP, Banerjee SS, Neavin D, Daouk RK, Rush AJ, Frye MA, Wang L, Weinshilboum R, Bobo WV, Iyer RK: Data-Driven Longitudinal Modeling and Prediction of Symptom Dynamics in Major Depressive Disorder: Integrating Factor Graphs and Learning Methods. in IEEE International Conference on Computational Intelligence in Bioinformatics and Computational Biology, IEEE Computational Intelligence Society; 2017.
